# Supplementary material for: Forward-looking serial intervals correctly link epidemic growth to reproduction numbers
Source: Proc Natl Acad Sci U S A. 2020 Dec 23;118(2):e2011548118. doi: 10.1073/pnas.2011548118 (PMC7812760; doi:10.1073/pnas.2011548118)
Supplement: Supplementary File [file pnas.2011548118.sapp.pdf]

Supporting Information

Forward-looking serial intervals correctly link epidemic growth to reproduction numbers

Sang Woo Park[1,*] Kaiyuan Sun[2] David Champredon[3] Michael Li[4] Benjamin M. Bolker[4,5,6] David J. D. Earn[5,6] Joshua S. Weitz[7, 8] Bryan T. Grenfell[1,2,9] Jonathan Dushoff[4,5,6]

1 Department of Ecology and Evolutionary Biology, Princeton University, Princeton, NJ, USA
2 Fogarty International Center, National Institutes of Health, Bethesda, MD, USA
3 Department of Pathology and Laboratory Medicine, University of Western Ontario, London, Ontario, Canada
4 Department of Biology, McMaster University, Hamilton, ON, Canada
5 Department of Mathematics and Statistics, McMaster University, Hamilton, ON, Canada
6 M. G. DeGroote Institute for Infectious Disease Research, McMaster University, Hamilton, ON, Canada
7 School of Biological Sciences, Georgia Institute of Technology, Atlanta, GA, USA
8 School of Physics, Georgia Institute of Technology, Atlanta, GA, USA
9 Woodrow Wilson School of Public and International Affairs, Princeton University, Princeton, NJ, USA

*Corresponding author: swp2@princeton.edu

Disclaimer: The findings and conclusions in this report are those of the authors and do not necessarily represent the official position of the U.S. National Institutes of Health or Department of Health and Human Services.

S1 Deterministic simulation

We simulate the renewal equation model using a discrete-time approximation:

$$i(t) = \mathcal{R}_0 S(t - \Delta t) \sum_{m=1}^{m_{\max}} i(t - m\Delta t)\hat{g}(m\Delta t)$$
$$S(t) = S(t - \Delta t) - i(t) \tag{S.1}$$

where $\hat{g}$ is a discrete-time intrinsic generation-interval distribution that satisfies the following:

$$\hat{g}(m\Delta t) = \frac{g(m\Delta t)}{\sum_{i=1}^{\ell} g(m\Delta t)}, \quad m = 1, \ldots, m_{\max}. \tag{S.2}$$

The continuous-time intrinsic generation-interval distribution is parameterized using a log-normal distribution (Table 1). We define the intrinsic incubation period distribution in a similar manner:

$$\hat{\ell}(m\Delta t) = \frac{\ell(m\Delta t)}{\sum_{i=1}^{\ell} \ell(m\Delta t)}, \quad m = 1, \ldots, m_{\max}, \tag{S.3}$$

where its continuous-time analog is also based on a log-normal distribution. For simplicity, we assume that the forward incubation periods and intrinsic generation intervals are independent:

$$\hat{h}(m\Delta t, n\Delta t) = \hat{\ell}(m\Delta t)\hat{g}(n\Delta t), \quad m, n = 1, \ldots, m_{\max}. \tag{S.4}$$

We use $\Delta t = 0.025$ days and $m_{\max} = 2001$ for discretization steps.

We initialize the simulation with population size N=40,000 as follows:

$$i(m\Delta t) = C \exp(rm\Delta t), \quad m = 1, \ldots, m_{\max}$$
$$S(m\Delta t) = N - \sum_{n=1}^{m} i(n\Delta t), \quad m = 1, \ldots, m_{\max} \tag{S.5}$$

where C is chosen such that $\sum_{n=1}^{m_{\max}} i(m\Delta t) = 10$. These initial conditions allow the model to follow exponential growth from time $\Delta t(m_{\max} + 1)$ without any transient behaviors.

S2 Stochastic simulation

We run stochastic simulations of the renewal equation model using an individual-based model on a fully connected network (i.e., homogeneous population) based on the Gillespie algorithm that we developed earlier (Park et al., 2020). First, we initialize an epidemic with $I(0)$ infected individuals (nodes) in a fully connected network of size N. For each initially infected individual, we draw number of infectious contacts from a Poisson distribution with the mean of $\mathcal{R}_0$ and the corresponding generation intervals for each contact from a log-normal distribution (Table 1). Contactees are uniformly sampled from the total population. All contactees are sorted into event queues based on their infection time. We update the current time to the infection time of the first person in the queue. Then, the first person in the queue makes contacts based on the Poisson offspring distribution described earlier and their contactees are added to the sorted queue. Whenever contactees are added to the sorted queue, we remove all duplicated contacts (but keep the first one) as well as contacts made to individuals that have already been infected. Simulations continue until there are no more individuals in the queue. We simulate 10 epidemics with $I(0) = 10$ and N=40,000.

S3 Linking r and $\mathcal{R}_0$ using serial-interval distributions

The intrinsic generation-interval distribution $g(\tau)$ provides a link between r and $\mathcal{R}_0$ via the Euler-Lotka equation (Wallinga and Lipsitch, 2007):

$$\frac{1}{\mathcal{R}_0} = \int_0^\infty \exp(-r\tau) g(\tau)\, \mathrm{d}\tau\,. \tag{S.6}$$

In this section, we prove that the initial forward serial-interval distribution $f_0(\tau)$ also estimates the same $\mathcal{R}_0$ from r, except that integral extends to $\tau = -\infty$ rather than beginning at $\tau = 0$, because serial intervals can be negative:

$$\frac{1}{\mathcal{R}_0} = \int_{-\infty}^\infty \exp(-r\tau) f_0(\tau)\, \mathrm{d}\tau\,. \tag{S.7}$$

Here, the initial forward serial-interval distribution $f_0(\tau)$ is defined as:

$$f_0(\tau) = \frac{1}{\phi} \int_{-\infty}^0 \int_{\alpha_1}^\tau \exp(r\alpha_1) h(-\alpha_1, \alpha_2 - \alpha_1) \ell(\tau - \alpha_2)\, \mathrm{d}\alpha_2\, \mathrm{d}\alpha_1\,, \tag{S.8}$$

where h is the joint probability distribution describing the intrinsic generation-interval distribution g and the intrinsic incubation period distribution ℓ (see Eq. (15) in the main text), and the normalization constant ϕ is determined by the requirement that $\int_{-\infty}^\infty f_0(\tau)\, \mathrm{d}\tau = 1$.

In order to verify Eq. (S.7), we first rewrite the integral in Eq. (S.8) by substituting $-\alpha_1$ for α_1, and then changing the order of integration:

$$\begin{aligned} f_0(\tau) &= \frac{1}{\phi} \int_0^\infty \int_{-\alpha_1}^\tau \exp(-r\alpha_1) h(\alpha_1, \alpha_2 + \alpha_1) \ell(\tau - \alpha_2)\, \mathrm{d}\alpha_2\, \mathrm{d}\alpha_1\,, \\ &= \frac{1}{\phi} \int_{-\infty}^\tau \int_{\max(0, -\alpha_2)}^\infty \exp(-r\alpha_1) h(\alpha_1, \alpha_2 + \alpha_1) \ell(\tau - \alpha_2)\, \mathrm{d}\alpha_1\, \mathrm{d}\alpha_2\,. \end{aligned} \tag{S.9}$$

To further simplify the expression, we define $z(\alpha_2)$ as follows:

$$z(\alpha_2) = \int_{\max(0, -\alpha_2)}^\infty \exp(-r\alpha_1) h(\alpha_1, \alpha_2 + \alpha_1)\, \mathrm{d}\alpha_1\,. \tag{S.10}$$

Substituting $z(\alpha_2)$ into Eq. (S.9) we obtain:

$$f_0(\tau) = \frac{1}{\phi} \int_{-\infty}^\tau z(\alpha_2) \ell(\tau - \alpha_2)\, \mathrm{d}\alpha_2\,,. \tag{S.11}$$

Writing $\hat{z}$ for a normalized version of z,

$$\hat{z}(\alpha_2) = \frac{z(\alpha_2)}{\int_{-\infty}^\infty z(x)\, \mathrm{d}x}\,, \tag{S.12}$$

we can now express the initial forward serial-interval distribution f_0 as a convolution of $\hat{z}$ and ℓ:

$$f_0(\tau) = \frac{1}{\hat{\phi}} \int_{-\infty}^{\tau} \hat{z}(\alpha_2)\ell(\tau - \alpha_2)\,\mathrm{d}\alpha_2\,, \tag{S.13}$$

where $\hat{\phi} = \phi / \int_{-\infty}^{\infty} z(x)\,\mathrm{d}x$.

Since the right hand side of Eq. (S.7) is also a Laplace transform of $f_0 = \hat{z} * \ell$, we can express it as the product of Laplace transforms of $\hat{z}$ and ℓ:

$$\int_{-\infty}^{\infty} \exp(-r\tau) f_0(\tau)\mathrm{d}\tau = \int_{-\infty}^{\infty} \exp(-r\tau)\hat{z}(\tau)\,\mathrm{d}\tau \int_0^{\infty} \exp(-r\tau)\ell(\tau)\,\mathrm{d}\tau\,. \tag{S.14}$$

In order to derive an expression for a Laplace transform of $\hat{z}$, we have to first derive an analytical expression for $\int_{-\infty}^{\infty} z(x)\,\mathrm{d}x$. By changing the order of integration, we have:

$$\begin{aligned}\int_{-\infty}^{\infty} z(\alpha_2)\mathrm{d}\alpha_2 &= \int_{-\infty}^{\infty} \int_{\max(0,-\alpha_2)}^{\infty} \exp(-r\alpha_1)h(\alpha_1, \alpha_2 + \alpha_1)\,\mathrm{d}\alpha_1\,\mathrm{d}\alpha_2\,, \\ &= \int_0^{\infty} \int_{-\alpha_1}^{\infty} \exp(-r\alpha_1)h(\alpha_1, \alpha_2 + \alpha_1)\,\mathrm{d}\alpha_2\,\mathrm{d}\alpha_1\,. \end{aligned} \tag{S.15}$$

Since ℓ is a marginal probability distribution of h, it follows that:

$$\int_{-\infty}^{\infty} z(\alpha_2)\mathrm{d}\alpha_2 = \int_0^{\infty} \exp(-r\alpha_1)\ell(\alpha_1)\,\mathrm{d}\alpha_1\,. \tag{S.16}$$

Then, we have:

$$\hat{z}(\alpha_2) = \frac{\int_{\max(0,-\alpha_2)}^{\infty} \exp(-r\alpha_1)h(\alpha_1, \alpha_2 + \alpha_1)\,\mathrm{d}\alpha_1}{\int_0^{\infty} \exp(-r\alpha_1)\ell(\alpha_1)\,\mathrm{d}\alpha_1}\,. \tag{S.17}$$

Substituting the expression into Eq. (S.14), we have:

$$\int_{-\infty}^{\infty} \exp(-r\tau) f_0(\tau)\,\mathrm{d}\tau = \int_{-\infty}^{\infty} \exp(-r\alpha_2) \int_{\max(0,-\alpha_2)}^{\infty} \exp(-r\alpha_1)h(\alpha_1, \alpha_2 + \alpha_1)\,\mathrm{d}\alpha_1\,\mathrm{d}\alpha_2\,. \tag{S.18}$$

Recall that g is also a marginal probability distribution of h:

$$g(\tau) = \int_0^{\infty} h(x, \tau)\,\mathrm{d}x\,. \tag{S.19}$$

We can then substitute $\tau = \alpha_1 + \alpha_2$ into Eq. (S.18) and apply change of variables to obtain:

$$\int_{-\infty}^{\infty} \exp(-r\tau) f_0(\tau)\,\mathrm{d}\tau \tag{S.20}$$

$$= \int_{-\infty}^{\infty} \exp(-r\alpha_2) \int_{\max(0,-\alpha_2)}^{\infty} \exp(-r\alpha_1)h(\alpha_1, \alpha_2 + \alpha_1)\,\mathrm{d}\alpha_1\,\mathrm{d}\alpha_2 \tag{S.21}$$

$$= \int_0^{\infty} \int_0^{\infty} \exp(-r\tau)h(\alpha_1, \tau)\,\mathrm{d}\alpha_1\,\mathrm{d}\tau \tag{S.22}$$

$$= \int_0^{\infty} \exp(-r\tau)g(\tau)\,\mathrm{d}\tau = \frac{1}{\mathcal{R}_0} \tag{S.23}$$

Therefore, the initial forward serial-interval distribution and the intrinsic generation-interval distribution give the same estimates of $\mathcal{R}_0$ from r. $\square$

S4 Comparing the estimates of $\mathcal{R}_0$ using the initial forward and the intrinsic serial-interval distributions

We use a simulation-based approach to compare the estimates of $\mathcal{R}_0$ based on the serial- and generation-interval distributions. To do so, we model the intrinsic generation-interval distribution and the incubation period using a multivariate log-normal distribution with log means μ_G, μ_I, log standard variances σ_G^2, σ_I^2, and log-scale correlation ρ; the multivariate log-normal distribution is parameterized based on parameter estimates for COVID-19 (Table 1). We construct forward serial intervals during the exponential growth period as follows:

$$F_i = -X_{1,i} + (G_i|X_{1,i}) + X_{2,i}, \tag{S.24}$$

where the backward incubation period $X_{1,i}$ of an infector is simulated by drawing random log-normal samples Y_i with log mean μ_I and log variance σ_I^2 and resampling Y_i, each weighted by the inverse of the exponential growth function $\exp(-rY_i)$; the intrinsic generation interval conditional on the incubation period of the infector $(G_i|X_{1,i})$ is drawn from a log-normal distribution with log mean $\mu_G + \sigma_G\rho(\log(X_{1,i}) - \mu_I)/\sigma_I$ and log variance $\sigma_G^2(1-\rho^2)$; the forward incubation period $X_{2,i}$ of an infectee is drawn from a log-normal distribution with log mean μ_I and log variance σ_I^2. We then calculate the basic reproduction number $\mathcal{R}_0$ using the empirical estimator:

$$\mathcal{R}_0 = \frac{1}{\frac{1}{N}\sum_{i=1}^{N}\exp(-rF_i)}. \tag{S.25}$$

We compare this with an estimate of $\mathcal{R}_0$ based on the intrinsic serial-interval distribution which has the same mean as the intrinsic generation-interval distribution (Svensson, 2007; Klinkenberg and Nishiura, 2011; Champredon et al., 2018; Britton and Scalia Tomba, 2019):

$$\mathcal{R}_{\text{intrinsic}} = \frac{1}{\frac{1}{N}\sum_{i=1}^{N}\exp(-rQ_i)}, \tag{S.26}$$

where

$$Q_i = -Y_i + (G_i|Y_i) + X_{2,i}. \tag{S.27}$$

S5 Applications: SEIR model

Consider a Susceptible-Exposed-Infectious-Recovered model:

$$\begin{aligned}\frac{\mathrm{d}S}{\mathrm{d}t} &= -\beta S I \\ \frac{\mathrm{d}E}{\mathrm{d}t} &= \beta S I - \gamma_E E \\ \frac{\mathrm{d}I}{\mathrm{d}t} &= \gamma_E E - \gamma_I I \\ \frac{\mathrm{d}R}{\mathrm{d}t} &= \gamma_I I \end{aligned} \tag{S.28}$$

where β is the transmission rate, $1/\gamma_E$ is the mean latent period, and $1/\gamma_I$ is the mean infectious period. We further assume that the latent period is equivalent to incubation period; in other words, infected individuals can only transmit after symptom onset. Then, the generation interval will be always longer than the incubation period.

The joint probability distribution of the intrinsic incubation periods and intrinsic generation intervals for this model can be written as:

$$h(x,\tau) = \begin{cases} 0 & x > \tau \\ \gamma_I \gamma_E \exp(-\gamma_I(\tau - x) - \gamma_E x) & x \leq \tau \end{cases} \tag{S.29}$$

Then, the intrinsic generation-interval distribution is given by:

$$\begin{aligned} g(\tau) &= \int_0^\tau h(x,\tau)\,\mathrm{d}x \\ &= \frac{\gamma_I \gamma_E}{\gamma_E - \gamma_I}(\exp(-\gamma_I \tau) - \exp(-\gamma_E \tau)) \end{aligned} \tag{S.30}$$

On the other hand, the initial forward serial-interval distribution is given by:

$$\begin{aligned} f_0(\tau) &\propto \int_{-\infty}^0 \int_0^\tau \exp(r\alpha_1) h(-\alpha_1, \alpha_2 - \alpha_1) \ell(\tau - \alpha_2)\,\mathrm{d}\alpha_2\,\mathrm{d}\alpha_1 \\ &\propto \int_{-\infty}^0 \int_0^\tau \exp(r\alpha_1) \exp(-\gamma_I \alpha_2 + \gamma_E \alpha_1) \exp(-\gamma_E(\tau - \alpha_2))\,\mathrm{d}\alpha_2\,\mathrm{d}\alpha_1 \\ &\propto \exp(-\gamma_E \tau) \int_{-\infty}^0 \int_0^\tau \exp((\gamma_E - \gamma_I)\alpha_2) \exp((r + \gamma_E)\alpha_1)\,\mathrm{d}\alpha_2\,\mathrm{d}\alpha_1 \\ &\propto (\exp(-\gamma_I \tau) - \exp(-\gamma_E \tau)) \int_{-\infty}^0 \exp((r + \gamma_E)\alpha_1)\,\mathrm{d}\alpha_1 \\ &\propto \exp(-\gamma_I \tau) - \exp(-\gamma_E \tau) \end{aligned} \tag{S.31}$$

Therefore, both the intrinsic generation intervals and the initial forward serial intervals are identically distributed and have the same mean.

S6 Simulations with correlated intrinsic incubation periods and intrinsic generation intervals.

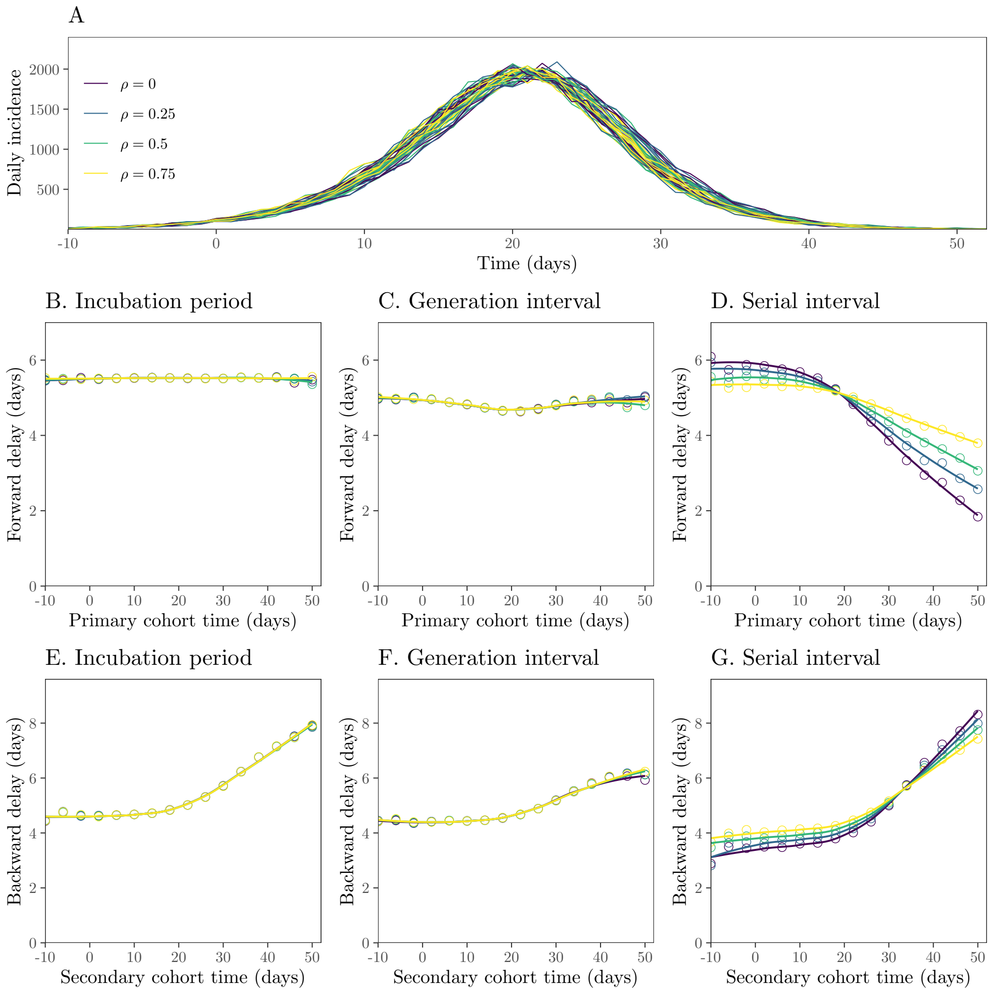

Figure S1: **Epidemiological dynamics and changes in mean forward and backward delay distributions.** (A) Daily incidence over time. (B–D) Changes in the mean forward incubation period, generation interval, and serial interval. (E–G) Changes in the mean backward incubation period, generation interval, and serial interval. Intrinsic incubation periods and intrinsic generation intervals are modeled using a correlated bivariate lognormal distribution; therefore, generation intervals are drawn from the corresponding conditional distributions (given a incubation period), instead of the marginal distribution. Higher correlation reduces the amount of changes in the mean forward serial interval because shorter (longer) backward incubation periods of infectors during the increasing (decreasing) phase of an epidemic are associated with shorter (longer) forward generation intervals. See Figure 3 in the main text for a detailed description.

References

Britton, T. and G. Scalia Tomba (2019). Estimation in emerging epidemics: Biases and remedies. *Journal of the Royal Society Interface 16*(150), 20180670.

Champredon, D., J. Dushoff, and D. J. D. Earn (2018). Equivalence of the Erlang-distributed SEIR epidemic model and the renewal equation. *SIAM Journal on Applied Mathematics 78*(6), 3258–3278.

Klinkenberg, D. and H. Nishiura (2011). The correlation between infectivity and incubation period of measles, estimated from households with two cases. *Journal of theoretical biology 284*(1), 52–60.

Park, S. W., D. Champredon, and J. Dushoff (2020). Inferring generation-interval distributions from contact-tracing data. *Journal of the Royal Society Interface 17*(167), 20190719.

Svensson, Å. (2007). A note on generation times in epidemic models. *Mathematical biosciences 208*(1), 300–311.

Wallinga, J. and M. Lipsitch (2007). How generation intervals shape the relationship between growth rates and reproductive numbers. *Proceedings of the Royal Society B: Biological Sciences 274*(1609), 599–604.
